# Social representation of spiritual surgeries in Umbanda: culture, religion and contributions of nursing theory

**DOI:** 10.1590/0034-7167-2022-0787

**Published:** 2023-12-04

**Authors:** Juliana de Lima Brandão, Antonio Marcos Tosoli Gomes, Laércio Deleon de Melo, Sergio Corrêa Marques, Gerson Lourenço Pereira, Renê dos Santos Spezani, Vívian Monteiro de Melo, Adriana da Silva Moço

**Affiliations:** I Universidade do Estado do Rio de Janeiro. Rio de Janeiro, Rio de Janeiro, Brazil; II Seminário Metodista César Dacorso Filho. Rio de Janeiro, Rio de Janeiro, Brazil.; III Centro Universitário Augusto Motta. Rio de Janeiro, Rio de Janeiro, Brazil; IV Universidade Santa Úrsula. Rio de Janeiro, Rio de Janeiro, Brazil; V Universidade Federal Fluminense. Niterói, Rio de Janeiro, Brazil

**Keywords:** Nursing Theory, Nursing Care, Culture, Religion, Social Psychology., Teoría de Enfermería, Atención de Enfermería, Cultura, Religión, Psicología Social., Teoria de Enfermagem, Cuidados de Enfermagem, Cultura, Religião, Psicologia Social

## Abstract

**Objective:**

to analyze the social representation of spiritual surgeries in Umbanda for Bantu-Amerindian ritual mediums and their contributions to the cross-cultural care proposed by Madeleine Leininger.

**Methods:**

a descriptive-exploratory, qualitative study, supported by the procedural approach of Social Representation Theory and Transcultural Nursing Theory, carried out with 30 Umbanda mediums of the Bantu-Amerindian ritual through interviews, submitted to the Iramuteq software for lexical analysis.

**Results:**

mostly women, white, with an average of 46 years old and approximately 14 years of practice in Umbanda participated. The social representation of spiritual surgeries in Umbanda is objectified and anchored through a biomedical vision of care, encompassing a set of beliefs, values and practices as religious treatments, through faith, whose main objective is healing.

**Final considerations:**

spiritual surgeries are a form of transcultural care, according to Madeleine Leininger’s propositions, as they integrate the culture of a group through health care in Umbanda.

## RESUMO

**Objetivo:**

analisar a representação social das cirurgias espirituais na Umbanda para médiuns do ritual bantu-ameríndio e suas contribuições para o cuidado transcultural proposto por Madeleine Leininger.

**Métodos:**

estudo descritivo-exploratório, qualitativo, apoiado na abordagem processual da Teoria das Representações Sociais e na Teoria do Cuidado Transcultural, realizado com 30 médiuns umbandistas do ritual bantu-ameríndio por meio de entrevistas, submetidas ao *software* Iramuteq para análise lexical.

**Resultados:**

participaram, majoritariamente, mulheres, brancas, na média dos 46 anos e aproximadamente 14 anos de prática na Umbanda. A representação social das cirurgias espirituais na Umbanda é objetivada e ancorada mediante uma visão biomédica do cuidado, englobando um conjunto de crenças, valores e práticas enquanto tratamentos religiosos, por meio da fé, cujo objetivo principal é a cura.

**Considerações finais:**

cirurgias espirituais são uma forma de cuidado transcultural, segundo proposições de Madeleine Leininger, por integrarem a cultura de um grupo através dos cuidados em saúde na Umbanda.

## RESUMEN

**Objetivo:**

analizar la representación social de las cirugías espirituales en Umbanda para médiums rituales bantú-amerindios y sus aportes al cuidado transcultural propuesto por Madeleine Leininger.

**Métodos:**

estudio descriptivo-exploratorio, cualitativo, sustentado en el enfoque procedimental de la Teoría de las Representaciones Sociales y la Teoría del Cuidado Transcultural, realizado con 30 médiums Umbanda del ritual Bantú-Amerindio a través de entrevistas, sometidos al software Iramuteq para análisis léxico.

**Resultados:**

participaron en su mayoría mujeres, blancas, con una edad promedio de 46 años y aproximadamente 14 años de práctica en Umbanda. La representación social de las cirugías espirituales en Umbanda se objetiva y ancla a través de una visión biomédica del cuidado, englobando un conjunto de creencias, valores y prácticas como tratamientos religiosos, a través de la fe, cuyo principal objetivo es la sanación.

**Consideraciones finales:**

las cirugías espirituales son una forma de cuidado transcultural según las proposiciones de Madeleine Leininger, en tanto integran la cultura de un grupo a través del cuidado de la salud en Umbanda.

## INTRODUCTION

Brazil is a country with a strong characteristic of cultural religiosity^(1)^, which includes *Umbanda*. It is an Afro-diasporic, syncretic, non-proselytizing, Christian, spiritualist, nature religion, practiced orally, made invisible by religious racism, whose foundation transits through mythical, historical and institutional accounts^(2)^. *Umbanda* also acts in situations of health and illness in different dimensions, being a reference for believers and consultants^(3-4)^. Thus, it is understood that spiritual surgery in *Umbanda*, a Bantu-Amerindian ritual, is part of health care, as it is a treatment or spiritual work, through faith, in which it is believed that energies are mobilized. The main objectives are to heal diseases, recover health, improve quality of life and strengthen faith^(5)^.

By itself, this practice reveals itself to be part of a system of beliefs shared among believers of a religious denomination, and evidences a certain degree of social belonging, in addition to demonstrating religious and cultural knowledge that transits through common sense. It serves as an option for health treatment, including for those who are not followers of the religion (consultants). Surgeries are scheduled and only occur with the participation of temple (*terreiro*) mediums. Patients lie on a stretcher, where mediums are around them, incorporated with their entities. The surgery is performed with magnetic hand position and, in some cases, *reiki* is used before, during and/or after. In most cases, it is not a single procedure, but a set of treatments that encompasses other religious practices simultaneously with biomedical treatment^(5)^.

Thus, a religious phenomenon is observed, but inherent in the field of health, which, due to its characteristics, can be investigated in the light of Social Representation Theory (SRT), according to Moscovici’s propositions^(6-7)^. Thus, SRT deals with investigating objects that are found in the field of social psychology, in which social representation (SR) is described as the understanding of common sense knowledge with a practical objective and influenced by the communication process^(8)^.

Moreover, it feeds the idea that, due to the natural complexity of issues involving health, care and its social actors, it is necessary to contemplate culture in the form of health care, preserving subjects’ subjectivity, i.e., their psychological characteristics^(9)^. This perspective is related to Madeleine Leininger’s Transcultural Nursing Theory, which deals with a way of planning and implementing health care that takes into account the cultural contribution of caregiver nurses and human beings who are cared for, respectfully and with dignity. This guarantees cross-cultural care that is congruent with multiple health demands^(10)^.

Therefore, spiritual surgery in *Umbanda* as well as other care provided by religion can be interpreted as a form of religious care aimed at the health of individuals or the community. An *Umbanda* temple, known as *terreiro*, can be understood as a health space, providing opportunities for research through phenomena of interest to science. Added to this are the knowledge gaps inherent in health rituals performed in a restricted manner, in addition to the natural difficulties in accessing them and participants, capable of being filled by the chosen theories^(5)^.

Therefore, the present study has its unique character, which brings innovations and advances to the knowledge already available for some reasons: 1 - there are no SR studies on spiritual surgeries published in scientific journals; 2 - there are very few studies on spiritual surgeries in *Umbanda* and, even if they exist, they do not have the focus intended here; 3 - about Bantu-Amerindian ritual, likewise, there is little literature available. On the other hand, it is one of the few *Umbanda* religious identities that practice spiritual surgeries and opened its space to researchers; 4 - as it is an initiatory religion based on orality, there is no literature common to *Umbanda* temples that deals with their practices; 5 - there is theoretical, methodological and philosophical robustness, with the use of two theories: one from the field of psychosociology and another from the health area, more specifically from nursing; and 6 - due to its dissemination and the space granted to a historically and culturally silenced religion, it allows raising possibilities for rescuing, historical repair, respecting and combating racism and religious intolerance.

Health and the health-disease process must be viewed holistically, with a focus on different social groups and sociocultural strata. The objective is to value popularized generic care, as it is socially shared, which presents patterns and practices of expressions of care that receive multiple influences. For example, there are religious and philosophical influences, cultural values, beliefs and ways of living^(11)^. This network of influences can be interconnected to spiritual surgeries as a representational object^(5,9)^, given its set of knowledge, beliefs, values and practices involved in the ritual, which must be respected and recognized by nursing professionals^(12)^.

Therefore, this study is highlighted in the face of the representational object in question, which transits in the sciences (health, social and religion) as well as the common sense that offers it support. It contributes in several ways, namely: thinking about new forms of health care comprehensively and culturally congruent; for the registration service provided to religions of African origin that work fundamentally with orality; and with science, transcending material barriers that prevent the observation of phenomena intrinsic to other paradigms^(5)^.

With this, we intend to answer the following guiding questions: what is SR of spiritual surgeries in *Umbanda*? How does this SR interrelate with transcultural care in the perspective proposed by Madeleine Leininger?

## OBJECTIVE

To analyze the SR of spiritual surgeries in *Umbanda* for Bantu-Amerindian ritual mediums and their contributions to the cross-cultural care proposed by Madeleine Leininger.

## METHODS

### Ethical aspects

The present study is part of a matrix investigation entitled “*Religiosidade e Espiritualidade em tempos de Covid-19: as implicações para a prevenção da infecção e o cuidado em saúde*”, approved by the Research Ethics Committee (REC). It met all ethical norms in research involving human beings, including the application of the Informed Consent Form (ICF) and recording of participants’ verbal consent in the interview. Moreover, only audio transcription was used, and participants’ secrecy, anonymity and voluntary character were ensured. With this, participants’ speeches are mentioned, being identified by “Participant 01” to “Participant 30”, followed by female or male.

### Theoretical-methodological framework

The study was carried out according to a theoretical-methodological triangulation, through the procedural approach^(13)^, of SRT^(6-7)^, and theoretical-philosophical, with Transcultural Nursing Theory, proposed by Madeleine Leininger^(10,14)^. The procedural approach, developed by Jodelet, emphasizes the constituent aspect of representations. It considers that access to knowledge of SR starts from the understanding of human beings as a producer of meanings, focusing on the analysis of symbolic productions, meanings and language, through which subjects construct the world in which they live^(13)^.

Thus, in the search for a nursing framework consistent with the sociocultural approach intended in this investigation, Madeleine Leininger’s proposal was identified as capable of supporting reflections by nursing and health professionals on health care, breaking taboos and prejudices in multidimensional care, including religious and spiritual care^(10-11)^. The epistemic convergences between Transcultural Nursing Theory and SRT are in the intersection provoked by culture in both theoretical sources. Culture is subsidized by values, norms, beliefs and ways of life of a group, which can be socially learned and shared, with the practical objective of guiding actions, decisions, thoughts and behaviors through standardized ways^(7,10,14)^.

The precepts of Leininger’s theory applied to the study are inserted in the three steps for professional practice in nursing care: a) preservation or cultural maintenance of care - with professional performance in supporting people as well as their training in favor of health preservation; b) accommodation or cultural negotiation of care - with help in activities related to the modes of negotiation, adjustments and health adaptation; c) repatterning or cultural restructuring of care - focusing on the adoption of healthier lifestyle habits, based on nursing assistance to modify lifestyle and health standards^(11)^. This means that, by knowing the SR of spiritual surgeries in *Umbanda*, nursing professionals can start to consider them as a form of care implemented from the culture of these religious people. Health is preserved by recognizing and supporting its own culture-based practice (spiritual surgery). By negotiation or accommodation, religious, biomedical or other treatments are adjusted, without disqualifying any. With restandardization, nursing can verify which actions to adopt to improve lifestyle and quality of health, respecting religious guidelines^(5)^.

### Study design

This is a descriptive and exploratory study, using a qualitative approach, due to the nature of the investigated phenomenon^(15)^. To this end, it was constructed respecting the checklist established by the COnsolidated criteria for REporting Qualitative research (COREQ) protocol, which safeguards the quality of qualitative studies^(16)^.

### Methodological procedures

Participants were accessed using the snowball technique as a way to gather the sample for convenience^(17)^. However, the technique was initially used to access the temples, when the research was presented, and then to the study participants. The collection instrument used was a questionnaire to investigate sociodemographic variables (sex, age, self-declared race/color and time practicing *Umbanda*). Then, individual in-depth interviews were carried out, with a semi-structured script, by the main researcher.

The interview questions aimed to find out about spiritual surgeries in *Umbanda* for the mediums in question, how they occur, what procedures were performed before, during and after, expected and obtained results, jointly oriented treatments and practices as well as any information about the experience of these mediums with the investigated health care practice.

### Study setting

Temples that include spiritual surgeries as a health care practice were selected for participation. More commonly, most of these centers practice a specific ritual: Bantu-Amerindian. Thus, a temple was invited to participate in the research, as it had the main researcher as a member for over 20 years, and indicated others who also practice the same ritual and perform spiritual surgeries. Altogether, three temples were selected, which are located in the Brazilian Southeast region: in Magé and Teresópolis, municipalities of Rio de Janeiro; and in Além Paraíba, in the countryside of the state of Minas Gerais.

It stands out as a relevant characteristic of the phenomenon studied in these scenarios, mainly the absorption of concepts inherent to the biomedical dimension of care, however through religious means. Secondly, the relevance of the entities present in Umbanda as “doctors” stands out, those who intercede for patients’ health. Finally, we highlight the absence of elements that, by analogy with physical surgeries, represent a surgical procedure, such as cutting and anesthetic induction^(5)^.

### Data source

A total of 30 people participated (10 from each temple), gathered by the snowball technique, self-declared mediums of *Umbanda*’s Bantu-Amerindian and who were ≥18 years old at the time of collection, indicated by the head of the temple, ho have been going to the temple for at least six months, and participated in at least one spiritual surgery in the temple where they work as a medium. People who left the temple during the collection period were excluded. Therefore, an *Umbanda* medium is understood to be one who works together with entities, in the context of *Umbanda* rituals, in a process known as incorporation, in which spiritual (non-living) entities work (magistic help) through mediums (alive)^(2)^.

### Data collection and organization

Data collection took place between August and September 2020, virtually, through Zoom^®^, due to the social distance required by the COVID-19 pandemic, as pointed out in another study^(18)^. The data from participant characterization were organized with the support of Microsoft Excel for Windows 365, and discursive content was transcribed into Microsoft Word for Windows 365 and later formatted to generate the final corpus.

### Data analysis

Participant characterization data was analyzed by simple statistics, such as frequencies and measures of central tendency, and the final *corpus* of the interviews was submitted to *Interface de R pour les Analyses Multidimensionnelles de Textes et de Questionnaires* (Iramuteq) to proceed with lexical analysis. This software promotes the analysis of textual content through lexicometry. Lexical analysis is mainly favorable when, when processing the data, categorical variables of context are inserted, such as situational characteristics of communication or enunciators, allowing to identify significant differences in the interviews considering the characteristics and groups to which participants belong. This possibility brings lexical analysis closer to content analysis. However, quantification is a possibility, not essential for content analysis, as it is for lexical analysis^(19)^. It can be used in many ways, being observed, in most studies, Reinert method or Descending Hierarchical Classification (DHC) use^(19-20)^, also used in the present research.

Iramuteq is anchored in another software, R, both free, through statistical processing. From there, Iramuteq starts lexical content analysis, processing the text units with the alteration of Initial Context Units (ICU), i.e., each interview, for Elementary Context Units (ECU): identifies the quantity and average frequency of words; analyzes words by their roots (textual reductions); creates a dictionary from reduced contents, detecting similar formats and additions or differences in classes^(20)^. With the division of classes carried out by the software’s statistical processing, speeches are arranged according to *corpus* homogeneity^(19)^. Therefore, when checking the statements for each class, they are renamed and analyzed, according to their textual content^(5)^.

## RESULTS

**Figure 1:**
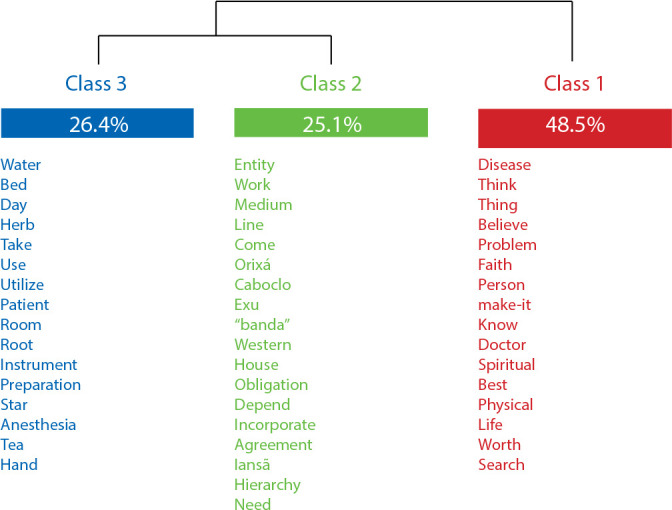
Descending Hierarchical Classification Dendrogram according to the interviews’ semantic contents. Rio de Janeiro, Rio de Janeiro, Brazil, 2022

*Source: extracted from Iramuteq, 2022.*

Study participants were mostly women (24; 80%), aged 46 years (variability 18 to 78), white (22; 73.33%) and *Umbanda* practitioners for approximately 14 years (5 to 47 variability). With regard to the lexical analysis, the interview *corpus* had an 87.84% use by Iramuteq, which identified 2,103 ECU, dividing the material into three classes, according to the dendrogram shown in Figure 1.

Thus, when grouping the semantic contents belonging to the classes, the material underwent an initial division, giving rise to class 1, “Spiritual surgeries: motives, expected results and faith”, and axis 1, “Social actors involved in spiritual surgeries: attributions, rituals and specificities of healing work”, which, in turn, was subdivided, originating classes 2, “Entities and mediums that work in spiritual surgeries”, and 3, “From preto post-surgery: procedures performed by entities, mediums and patients”, ending cluster analysis.

### Class 1 - Spiritual surgeries: motives, expected results and faith

It comprised 48.45% (1,019 of 2,103) of the ECU identified by the software, being the largest class due to its homogeneity. Disease (x2: 82.85), find (x2: 82.84), thing (x2: 78.48), believe (x2: 61.55), problem (x2: 50.18), faith (x2: 47.56), person (x2: 46.35), achieve (x2: 43.92), know (x2: 41.59), doctor (x2: 40.54), among others, were the main elements. It contextualized the spiritual surgery in *Umbanda* of Bantu-Amerindian ritual with motives, conditions and justifications:

*Spiritual surgery comes as an option to have this treatment, since, suddenly, material medicine has not been able to fully contemplate that disease, that illness or that problem that that person is going through at that moment.* (Participant 07, female, x^2^: 369.71)

*For spiritual surgery, you do have to have a physical problem, but much of what is there has something spiritual behind it, that’s why you need to treat the spirit so that the physical body is cured of that problem that is being worked on at the time of surgery.* (Participant 27, male, x^2^: 217.67)

The role of faith as fuel to achieve the intended result was evidenced:

*In our ritual, I’ve seen a lot of things happen, many people improve in terms of health, but I’m sure that those people who managed to reach their goal had faith, because, without faith, they wouldn’t have been able to reach their goal.* (Participant 27, male, x^2^: 323.58)

Moreover, the expected results of these spiritual surgeries were expressed:

*He always hopes for a positive result, that the person’s problem will end and that he will have, from then on, a life with a better quality than he presented* [...]. (Participant 21, female x^2^: 300.26)

*In reality, due to my great credulity, I hope that the disease itself will be extirpated, however, as I said, there are diseases that are not completely spiritual, they do not manage to go away in general.* (Participant 16, male, x^2^: 265.38)

### Axis 1 - Social actors involved in spiritual surgeries: attributions, rituals and specificities of healing work

This axis contemplated the role of each social actor involved, bringing classes 2 and 3.

### Class 2 - Entities and mediums that work in spiritual surgeries

Relative to 25.11% (528 of 2,103) of the ECU, it presented entity (x2: 319.34), work (x2: 152.61), medium (x2: 104.2), line (x2: 70.25), come (x2: 63.4), *orixá* (x2: 53.97), *caboclo* (x2: 51.54), *exu* (x2: 49.44), *banda* (x2: 46.42), Western (x2: 43.03), among others, as the main elements. This class addressed the main entities and mediums who work in spiritual surgeries.

As main entities in spiritual surgeries, without restriction, it was observed:

*Inside our house, we work specifically with Westerns, but we have some Umbanda entities, such as the caboclos line, which are entities that also work with healing.* (Participant 01, female, x^2^: 694.40)

*Westerns, within our Umbanda, are entities that are linked to the East, to the lands of the East. They are linked to entities that bring a lot of wisdom, do healing work, work with this organic regeneration and work with the 4 elements.* (Participant 01, female, x^2^: 613.10)

*For example, we use an iansã. We have cases, for instance, of rheumatism, cases that get worse, like lupus, which can be treated with a nana. So, it depends, each case is a specific entity type.* (Participant 08, female, x^2^: 576.90)

There were criteria to be observed for mediums to participate in surgeries. Furthermore, it was noted the insertion of Integrative and Complementary Health Practices (ICHP), such as *reiki*:

*From the head obligation, he already knows the medium’s “banda”, so they already gave signs that he has oriental entities in the “banda”. So, in a clear need, to complement the current of the group, they can be called, but, in principle, the most developed mediums, the oldest ones.* (Participant 01, female, x^2^: 626.38)

*The entity responsible for the surgery comes, sees the problem and performs surgery. Afterwards, mediums incorporate, then, as soon as all this is over, Reikian mediums go, help people who are guiding that stretcher to do reiki.* (Participant 21, female, x^2^: 535.02)

### Class 3 - From preto post-surgery: procedures performed by entities, mediums and patients

It expressed 26.44% (556 of 2,103) of ECU and had water (x2: 84.7), bed (x2: 68.63), day (x2: 63.13), herb (x2: 62.1), take (x2: 60.76), use (x2: 57.83), utilize (x2: 55.13), patient (x2: 52.35), room (x2: 44.89), among others, as main elements. The last class presented specific procedures performed by entities, mediums and patients before, during and after surgery.

It was noted that spiritual surgery had procedures to be fulfilled previously:

*These herbs are kept under that person’s bed all the time and then, at the end, we take this herb, keep it and deliver it to the consultant, who will make a bath in a certain number of days.* (Participant 02, female, x^2^: 356.87)

*As soon as it arrives, there is a reiki tea that is made with star anise, lemongrass and lemongrass, I think. And we give them to drink, they clean the stretchers, as if it were a reiki, and ask the consultant to lie down.* (Participant 21, female, x^2^: 222.89)

*Just the bath with herbs, the firmness of a guardian angel and the normal protection. It’s like a session. They are usually asked to participate in reiki first. There is also preparation with a bath with herbs on the day.* (Participant 21, female, x^2^: 221.92)

*Usually, they ask for some kind of specific exam in that area that will be done there, during the surgery process. If they brought it, they also confirm everything before starting. Guidance is given to all mediums regarding the surgery* [...]. (Participant 27, male, x^2^: 219.45)

In the same way, as a transoperative, during the spiritual surgery, there were procedures:

*He uses alcohol to wash his hands and the candle fire. We have a candle next to him, which catches that fire and goes on, doing it as if it were a cauterization, and, sometimes, in the final part of the surgery, but there is no object.* (Participant 02, female, x^2^: 329.44)

*The crystal is used and no other object is used. It’s with your hands. The patient or consultant has no cut and no bleeding. On average, there are 6 mediums incorporated per litter.* (Participant 24, male, x^2^: 301.78)

*It has a blue light, very calm. The person is always with the same amount of soft light and there is also a table that we use for fluidized water.* [...] (Participant 02, female, x^2^: 274.55)

*The work begins, we get mediums, first, to be able to perform anesthesia through magnetic hand position* [...]. (Participant 08, female, x^2^: 242.99)

At the end of surgery, other procedures and instructions were performed and provided:

*They leave the spiritual surgery room and take home a bottle of fluidized water, one laced with herbs that are selected by the temple, and they, from there, for 7 days, take baths with herbs and make use of fluidified water.* (Participant 09, female, x^2^: 265.73)

*There is a ritual that Dr. João does, that the material he uses on the consultant is a tissue, a gauze, it is all burned at each surgery*. [...] (Participant 26, female, x^2^: 240.91)

*Herbal baths, pulling, all that, and depending on fire work. There is reiki, then, leaving a room, going to another* [...]. *Every spiritual surgery, we ask for 3 days of rest, not to gain weight, these things.* (Participant 20, female, x^2^: 224.26)

## DISCUSSION

Participant characterization data reflects female empowerment as matripotence in religion^(21-22)^, in addition to drawing attention to the fact that they are white. This racial issue reinforces the historical-religious movement of whitening *Umbanda* in southeastern Brazil, with the intention of achieving the title of a genuinely Brazilian religion and erasing the black contributions present in *macumbas* in Rio de Janeiro^(23)^. This reflects the structural and religious racism in religions of African origin and in Brazil, and their links with colonialism, as they prevent relevance and recognition of black African heritage, encouraging violence and religious intolerance.

As for SR, it is emphasized that they respond to four essential functions: knowledge (allow understanding and explaining reality); identity (situating the actors in a social field); guidance (guides behaviors and practices); and justification (explain the behaviors and social behaviors)^(24)^. From the SR of spiritual surgeries, these are understood as a form of complementary treatment, through alternative ways, especially when patients no longer find results in the official system, but aim for a better quality of life. This has already been evidenced by the Cancer Patient Support Center (CAPC - *Centro de Apoio ao Paciente com Câncer*), in Florianópolis, Brazil^(25)^.

Spiritual surgeries emerge as a form of care, in the spiritual realm, structured on people’s faith^(5)^, and add to the care provided by health and nursing professionals^(26)^. However, there is a double understanding about faith, because, for *Umbanda* practitioners, if a healing practice is not successful, there are three possible meanings: lack of merit; lack of faith; or due to karmic actions. On the other hand, if the goal, especially that of healing, is achieved, it serves as a strengthening of faith^(27)^. In one way or another, spiritual surgery in *Umbanda* is closely linked to faith as a human action, whether in the Divine or in the potential of human beings themselves.

It should be noted that, from a holistic perspective of the health-disease process, care standards and practices are (in)directly influenced by factors, such as cultural values, beliefs and ways of life as well as religiosity, spirituality and personal life philosophy. Then, faith and religious practices gain prominence as elements of cross-cultural care^(10,14)^, showing that these anchor the group’s SR. This happens through SR’s formation process, which intends to make the unfamiliar familiar, as objectification transforms something that is abstract into concrete, and anchoring mobilizes pre-existing knowledge to incorporate something new and strange, making it familiar^(7)^. Thus, faith and hope justify the search of believers for spiritual surgeries, manifesting the functions of orientation and justification through the evaluative or affective representational dimension, because hope in healing, fueled by faith in the search, subsidizes the attitude adopted: going to the temple and undergoing spiritual surgery^(5)^.

When turning their gaze to *Umbanda* entities, participants cited Easterns, *Caboclos, Pretos-Velhos, Nanã, Iansã* and *Exu* acting in spiritual surgeries, each within their domain. As for the insertion of mediums in surgeries, they also need preparation. In this way, the functions of knowledge and identity are expressed through SR’s cognitive and informative (knowledge necessary for the act) and imagery (entities) dimensions. *Umbanda*, then, can be understood as “a popular health equipment” different from the biomedical model, as its characteristics represent a model of comprehensiveness, acceptance and humanization^(28)^. Thus, it is necessary to think about the health care of this subject, including their own references.

However, health models interact with each other in these temples (therapeutic complementarity) with some ICHP, such as *reiki*, chromotherapy and medicinal plants, expressing the objectification of SR of spiritual surgeries in *Umbanda* for the group. These therapies have been provided by the Brazilian Health System (SUS - *Sistema Único de Saúd*e) since 2006, with the Brazilian National Policy on Integrative and Complementary Practices (PNPIC - *Política Nacional de Práticas Integrativas e Complementares*)^(29)^. Thus, *reiki* is understood as a natural means aimed at prevention and cure, especially in the context of balance and harmony of mental, physical, emotional, spiritual and energetic health^(30)^. Therefore, one can interpret its role as a potentiator of spiritual surgery. In addition to these, other elements were used, such as fluidized water, alcohol, candle, crystal, which represent the imagery dimension, mainly according to the sociocognitive process of objectifying SR.

It should also be noted that there is no cut or anesthetic medication in this type of surgery, where everything is done with spiritual and magnetic hand position^(25)^. These symbolic and representative procedures in the spiritual-religious context portray a form of popular health care within the scope of spiritual surgeries in *Umbanda*. However, although the investigated universe presents its own conception of health care through spiritual surgeries, many aspects are crossed by the biomedical language as well as procedures (burned gauze after use) and post-surgical guidelines with the same pattern, when asking patients to rest, as the name itself: surgery.

This is the state of cognitive polyphasia, described by Moscovici^(6)^ and deepened in another work^(31)^, in which, according to SRT, the same social object can have different meanings coexisting, considering the numerous identities that people have, in the context of different social groups, and because they transit in different scenarios. In this way, representational fields have different means of thinking concomitantly in the same social group and even before the same individual due to cognitive polyphasia. Furthermore, the states of cognitive polyphasia demonstrate the connections between SR, culture and identity, and, with that, ratify the plural internal logic inherent to human beings’ sociocognitive systems^(31-32)^.

Thus, healing care practices are made possible, among other ways, through spiritual surgeries^(5)^, in a process of repatterning, through cultural restructuring of care^(14)^, from incorporation of knowledge and religious practices popularly anchored in biomedical care. This process is called assimilation, in which new information is inserted in individuals’ cognitive structure, through the adaptive effort, changing the acquired schemes and adapting to a new content based on a pre-existing mental structure^(11)^.

Moreover, patients also have other forms of religious treatment, such as “pulling”, which are spiritual cleansings to remove people’s negative energies^(33)^, and “fire works”, where a dot is scratched on the ground, covered in gunpowder, whose objective is to help people^(34)^. Dot scratched on the ground is a form of graphics in *Umbanda* with the ability to interconnect the spiritual and terrestrial planes, in addition to identifying the entities^(35)^. In this way, accommodation, or even cultural negotiation of care^(14)^, occurs through different interventions and religious care, along with spiritual surgeries in *Umbanda* and the hegemonic medical model.

Still with regard to cross-cultural care, it is possible to understand where *Umbanda* is inserted, because, according to the Sunrise Model, in the upper half, there are the social structures that guide people’s world view, through their culture, and on the horizontal base, there are the popular and professional systems that, in turn, are influenced by the above structure. With this, it is understood that the “sun” demonstrates the Bantu participation guided by *Umbanda* and its origin, and on the horizon is the SUS, its services and support network. Thus, an *Umbanda* temple integrates a popular religious health system in the horizon of the model influenced by upper structures^(5)^.

The Sunrise Model is composed of four levels: I - responsible for the world view and social perspectives; II - includes individual or family group explanations, added to health care knowledge and practices; III - considers traditional and professional knowledge and its insertion in the cultural context, enabling the identification of diversity as well as universality in cultural care; and IV - points out decisions related to the care practiced by nursing, which include accommodation, preservation and repatterning according to cultural care. This is where culturally coherent care is delivered^(10)^.

Thus, in the upper half of the Sunrise Model, level I is composed of technological, religious and physiological, kinship and social, social values and ways of life, political and legal, economic and educational factors. This set exerts a direct influence on the patterns and expressions of care in health and well-being. Level II, enclosing the upper half, is composed of subjects, families and institutions, which make up the different health systems. Religious groups, with their knowledge, their practices, their worldview and their SR, are part of a support network within a popular system that, together with professional systems, ensures culturally coherent care^(10)^.

Thus, it is noted that, once aware that an SR is common sense knowledge with practical purposes, influenced within communications and by culture^(8-9)^, among other factors, the Sunrise Model, aimed at contextualization of cultural care, it contributes by exemplifying that the path of formation of this care coincides with the set that gives body to the SR of a practice of religious health care encompassed therein, mainly for demonstrating the relevance of the dimensions of the cultural and social structures that support them. Cross-cultural care must, therefore, pay attention to cultural diversity and the vision of health and illness that individuals have, in order to effectively interfere with their reality^(36)^. This reflects the importance that religion and healing rituals have for these believers and social actors, which needs to be respected and naturalized^(37)^.

### Study limitations

Despite having accessed participants from temples located in three cities in two states of Brazil, it is understood to be a limitation of this study not having reached a larger number. Furthermore, this is a study with an unprecedented character, which implies scarce literature for dialogue.

### Contributions to nursing, health, or public policies

The present study contributes by acting as a practical example of transcultural care, observing the propositions of Madeleine Leininger’s Transcultural Theory, in which culture is expressed and interrelates with religion through religious health care that transit in believers’ common sense and are present in health care structures. In addition to this, there is a relevant record of religious practice passed down through generations through orality.

## FINAL CONSIDERATIONS

The SR of spiritual surgeries in *Umbanda* for *Umbanda* mediums of the Bantu-Amerindian ritual proves to be a practice of religious health care in *Umbanda*, guided by a set of beliefs and values shared by its believers, which aims at healing, recovering health, or improving quality of life as a form of complementary treatment. In addition, as religious and common-sense knowledge, it is crossed by the biomedical dimension, which is attributed to cognitive polyphasia. In this way, it can be understood as a form of transcultural care, according to Madeleine Leininger’s propositions, when understanding that it is present in the culture of a people or a portion that identifies with Umbanda. It is understood, therefore, that it contributes to comprehensive health care, respecting the values and beliefs of human beings who need care and verifying in them a complementary opportunity.
